# Retraction: Psychological support for public-funded normal students engaged in teaching profession

**DOI:** 10.3389/fpsyg.2026.1875898

**Published:** 2026-05-15

**Authors:** Frontiers Editorial Office

The journal retracts the 29 July 2022 article cited above.

Following publication, concerns were raised regarding the validity of the data in the article. The authors failed to provide a satisfactory explanation during the investigation, which was conducted in accordance with Frontiers' policies. Given the concerns, and the lack of raw data, the editors no longer have confidence in the findings presented in the article.

This retraction was approved by the Chief Editors of Frontiers in Psychology and the Chief Executive Editor of Frontiers. The authors received a communication regarding the retraction and had a chance to respond. This communication has been recorded by the publisher.

